# Prokaryotic Diversity and Composition of Sediments From Prydz Bay, the Antarctic Peninsula Region, and the Ross Sea, Southern Ocean

**DOI:** 10.3389/fmicb.2020.00783

**Published:** 2020-04-28

**Authors:** Jiang Li, Xiaoqian Gu, Yuanyuan Gui

**Affiliations:** ^1^Marine Bioresource and Environment Research Center, First Institute of Oceanography, Ministry of Natural Resources, Qingdao, China; ^2^Ministry of Natural Resources (MNR) Key Lab for Science & Technology of Marine Ecosystems, First Institute of Oceanography, Ministry of Natural Resources, Qingdao, China; ^3^College of Environmental Science and Engineering Qingdao University, Qingdao, China

**Keywords:** prokaryotic diversity, 16S rRNA gene, geophysicochemical factors, high-throughput sequencing (HTS), the Southern Ocean

## Abstract

The V3–V4 hypervariable regions of the 16S ribosomal RNA gene were analyzed to assess prokaryotic diversity and community compositions within 19 surface sediment samples collected from three different regions (depth: 250–3,548 m) of Prydz Bay, the Antarctic Peninsula region, and the Ross Sea. In our results, we characterized 1,079,709 clean tag sequences representing 43,227 operational taxonomic units (OTUs, 97% similarity). The prokaryotic community distribution exhibited obvious geographical differences, and the sequences formed three distinct clusters according to the samples’ origins. In general, the biodiversity of Prydz Bay was higher than those of the Antarctic Peninsula region and the Ross Sea, and there were similar prokaryotic communities in different geographic locations. The most dominant clades in the prokaryotic communities were Proteobacteria, Bacteroidetes, Thaumarchaeota, Oxyphotobacteria, Deinococcus-Thermus, Firmicutes, Acidobacteria, Fusobacteria, and Planctomycetes, but unique prokaryotic community compositions were found in each of the sampling regions. Our results also demonstrated that the prokaryotic diversity and community distribution were mainly influenced by geographical and physicochemical factors, such as Zn, V, Na, K, water depth, and especially geographical distance (longitude variation of sample location) and Ba ion content. Moreover, geochemical factors such as nutrient contents (TC, P, and Ca) also played important roles in prokaryotic diversity and community distribution. This represents the first report that Ba ion content has an obvious effect on prokaryotic diversity and community distribution in Southern Ocean sediments.

## Introduction

Antarctica is arguably the world’s most important continent for influencing the Earth’s climate and ocean ecosystem function (Wilkins et al., 2012). Upwelling of nutrient-rich Circumpolar Deep Water (CDW) returns nutrients transported to the deep ocean by the sinking of organic matter and supports 75% of global ocean primary production north of 30°S. The unique physicochemical properties of the Southern Ocean enable high levels of microbial primary production to occur (Wilkins et al., 2013), however, most of its microbial communities remain unexplored. Microorganisms are fundamental to the functioning of Antarctic ecosystems; in addition, the species diversity in specific habitat and differences in niches between habitats are fundamental ecological questions.

An increasing number of studies have recently been carried out on bacterial communities in different geographical regions of Antarctica and the Southern Ocean (Aislabie et al., 2008; Chong et al., 2009a, b, 2013; Shivaji et al., 2011; Huang et al., 2013; Anders et al., 2015; Siddarthan et al., 2019). Most of these studies have focused on the bacterial diversity and function of soils, lakes, surface seawater and sea ice (Yergeau et al., 2007a, b, c, 2009, 2012; Yergeau and Kowalchuk, 2008; Anders et al., 2015; Julie et al., 2017). However, relatively few studies have been conducted on prokaryotic diversity in sediments, especially deep-sea sediments, from the Southern Ocean using high-throughput sequencing (HTS) technology. Two reports focusing on mesopelagic sediments (150–1,000 m) collected from the Eastern sector of the Southern Ocean have been published (Bowman and McCuaig, 2003; Bowman et al., 2003), and studies of archaeal diversity in Antarctic sediments of shallow coastal environments have been performed (Bowman et al., 2000; Powell et al., 2003; Purdy et al., 2003). In recent years, increasing evidence based on large-scale spatial comparisons has revealed far greater complexity in the biogeographic patterns of terrestrial ecosystems of Antarctica than was previously appreciated (Chown and Convey, 2007; Convey et al., 2008; Terauds et al., 2012). These studies showed that strongly localized diversity was detected when comparing the genetic lineages of Antarctic microbial eukaryotic organisms across different locations (Lawley et al., 2004; Namsaraev et al., 2010). Moreover, a study by Dong et al. (2017) demonstrated that bacterial distribution was significantly correlated with sediment geophysicochemical factors. However, knowledge about the relationship between both the diversity and distribution of prokaryote in surface sediments and geophysicochemical factors of sediments in the Southern Ocean remain limited.

The Southern Ocean (SO) has been an area of particular biogeochemical interest because of the presence of macronutrients (N, P, and Si) (Martin et al., 1990; Cassar et al., 2007; Moore et al., 2013). Prydz Bay exhibits the largest shelf on the eastern margin of Antarctica (Harris et al., 1998). It receives sediments supplied by the Lambert Glacier/Amery Ice Shelf, draining approximately 10% of the total ice volume of Antarctica (Forsberg et al., 2008). The Ross Sea is one of the most productive areas of the Southern Ocean and includes several functionally different marine ecosystems. Limited analysis of Southern Ocean sediments and deep water has been performed, the majority of those studies focusing on surface waters.

The aim of the present work was to investigate the prokaryotic diversity and distribution in 19 sites (250–3,548 m depth) of Prydz Bay, the Antarctica Peninsula region, and the Ross Sea using HTS technology. The three regions are quite different geographically and climatically, and regional and local variability may have important influences on the prokaryotic diversity and function of these Antarctic ecosystems. Understanding the spatial scales at which these ecosystems operate provides a basis for conservation planning and exploitation of microbial resources in Antarctica. To our knowledge, comparative analysis of the prokaryotic diversity and composition of sediments from Prydz Bay, the Antarctica Peninsula region, and the Ross Sea has never been investigated using this approach.

## Materials and Methods

### Site Locations and Sample Collection

**FIGURE 1:**
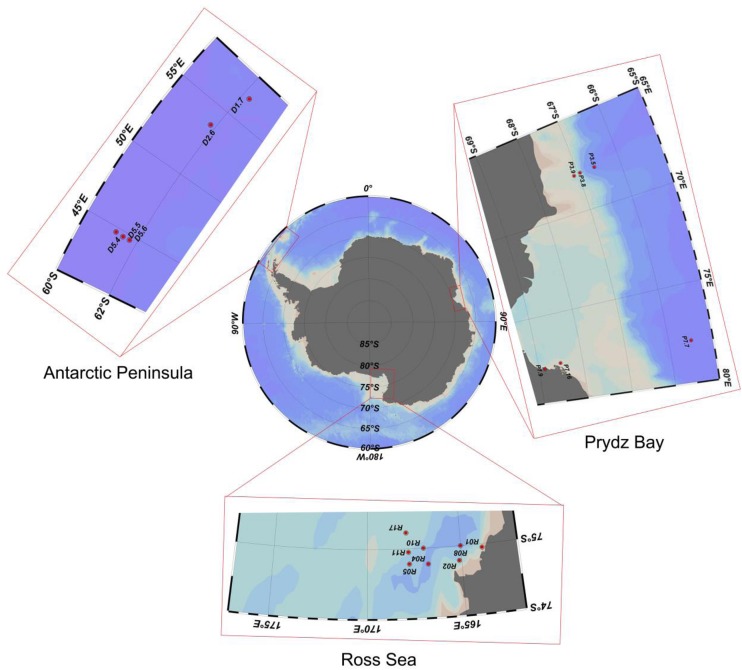
Sampling sites in three regions of the Southern Ocean.

Sediment samples were collected by the Icebreaker “*Xuelong*” from Prydz Bay (6 sites, average depth 1537 ± 1083 m), the Antarctic Peninsula (5 sites, 1085 ± 1390 m), and the Ross Sea (8 sites, 634 ± 193 m) during China’s Antarctic scientific investigation from 2013 to 2015 (Figure 1). The sediments were sampled by a multicore sampler (Ø 10 × 60 cm for each core sampler), 100 g sediment deposits were randomly collected from three different sites of uppermost layers (depth of 0–2 cm) with a sterile spoon and immediately transferred to sterile plastic tubes (Table 1). All the samples were stored in a −80°C freezer on board until further analysis.

**TABLE 1:** Sample sites description of the 19 surface sediment samples from three regions of the Southern Ocean.

Sampling region	Sampling site name	Sampling site description						
		Longitude	Latitude	Layer (cm)	Collected Method	Sample amount (g)	Water depth (m)	Collected time
Prydz Bay	P3.5	67°56′09^″^E	66°36′02^″^S	0–2	Multicore sampler	100	2064	2013-02-02
	P3.8	67°57′33^″^E	66°57′16^″^S	0–2	Multicore sampler	100	1487	2013-03-02
	P3.9	68°00′03^″^E	67°06′12^″^S	0–2	Multicore sampler	100	250	2013-03-02
	P7.7	77°53′04^″^E	65°29′10^″^S	0–2	Multicore sampler	100	3250	2013-01-31
	P7.9	78°02′21^″^E	68°52′06^″^S	0–2	Multicore sampler	100	1628	2013-02-02
	P7.16	77°50′40^″^E	68°32′06^″^S	0–2	Multicore sampler	100	540	2013-02-03
Antarctic Peninsula	D2.6	53°42′02^″^W	61°49′17^″^S	0–2	Multicore sampler	100	810	2014-02-10
	D1.7	56°34′41^″^W	62°15′43^″^S	0–2	Multicore sampler	100	3548	2014-02-08
	D5.4	44°41′44^″^W	61°12′00^″^S	0–2	Multicore sampler	100	317	2014-02-14
	D5.5	44°39′30^″^W	61°29′38^″^S	0–2	Multicore sampler	100	366	2014-02-14
	D5.6	44°40′32^″^W	61°44′23^″^S	0–2	Multicore sampler	100	386	2014-02-14
Ross Sea	R01	163°52′05^″^E	74°56′49^″^S	0–2	Multicore sampler	100	516	2014-12-30
	R02	165°7′58^″^E	74°47′06^″^S	0–2	Multicore sampler	100	719	2014-12-30
	R04	166°46′36^″^E	74°46′00^″^S	0–2	Multicore sampler	100	901	2014-12-31
	R05	167°46′39^″^E	74°46′36^″^S	0–2	Multicore sampler	100	586	2014-12-31
	R08	165°00′43^″^E	75°00′10^″^S	0–2	Multicore sampler	100	892	2015-01-03
	R10	167°00′07^″^E	74°59′68^″^S	0–2	Multicore sampler	100	636	2014-12-31
	R11	167°48′20^″^E	74°56′57^″^S	0–2	Multicore sampler	100	449	2014-12-31
	R17	167°54′33^″^E	75°13′49^″^S	0–2	Multicore sampler	100	374	2015-01-05

### Geochemical Analysis of Sediments

The total carbon (TC), total organic carbon (TOC), and total nitrogen (TN) contents of each homogenized sediment sample dried by vacuum refrigeration were determined by a Vario EL III Elemental Analyzer (Elementar Analysensysteme GmbH, Hanau, Germany) at the Laboratory of Marine Sedimentology and Environmental Geology, Ministry of Natural Resources. Subsequently, the Ba, Al, Fe, Mg, Ca, K, Na, Mn, Ti, V, Zn, and P contents were measured with an X-ray fluorescence spectrometer (Axios) according to the method of Dong et al. (2017). In detail, each sediment (5 g) was freeze-dried for 48 h, mechanically ground with a carbon–tungsten alloy mill and then crushed into a sheet (40 mm in outer diameter and 30 mm in inner diameter) under 20 tons of pressure. Finally, the sheets were used for element analysis.

### Environmental DNA Extraction and PCR Amplication

Total genomic DNA was extracted from each homogenized sediment sample (0.5 g) using a FastDNA Spin Kit for Soil (MP Biomedicals, Santa Ana, CA, United States) following the manufacturer’s instructions. The extracted DNA samples were quantified using a NanoDrop 2000 spectrophotometer (Thermo Fisher Scientific, Waltham, MA, United States).

The conserved sequences flanking the hypervariable V3–V4 region of the rRNA gene served as primer sites to generate PCR amplicons. PCR was performed on 20 ng of sample DNA using a PCR mix containing 0.2 μl *Taq* polymerase, 5 μl buffer, and a 10 μM concentration of both forward and reverse primers 341F (CCTAYGGGRBGCASCAG) and 806R (GGACTACNNGGGTATCTAAT) with Barcode Tags. The amplicon libraries were constructed under the following PCR conditions: 94°C for 2 min, and 30 cycles of 94°C for 30 s, 55°C for 20 s, and 72°C for 1 min, with a final extension step held at 72°C for 10 min. Thereafter, amplicon PCR products were purified using QIA quick PCR purification columns (Qiagen Inc., Valencia, CA, United States) and pooled in equimolar concentrations. The amplicon library was purified and sequenced on an Illumina MiSeq sequencing-by-synthesis (SBS) platform (Novogene Bioinformatics Technology Co. Ltd, Beijing, China).

### Sequence Data Analysis

After sequencing, the barcodes and amplicon primer sequences were removed, and reads with more than one unknown nucleotide (N), reads with ≥3% of bases with Phred values of <27, and reads with a length greater than 2 standard deviations away from the mean read length were removed. Then the chimeras of reads were removed using UCHIME Algorithm. Subsequent analyses on the resulting pooled reads were carried out using QIIME (V1.7.0). Pooled sequences were denoised using Acacia (version 1.5), which algorithmically corrected pyrosequencing errors and removed reads with a length more than 2 standard deviations away from the mean read length. The operational taxonomic units (OTUs) was clustered at 97% similarity using the uclust OUT picking method, the most-abundant reads from each OTU were aligned using the Py-NAST algorithm. Alpha diversity was applied in analyzing the complexity of species diversity for each sample, and the observed-species, Chao1, Shannon, Simpson, ACE, and Good’s coverage indices were evaluated using the QIIME software (V1.7.0) (Gregory et al., 2010). Principal coordinate analyses (PCoA) based on weighted and unweighted UniFrac distance (Lozupone and Knight, 2005) were conducted to investigate the differences in prokaryotic community structures between the sampling sites (Catherine et al., 2011). A Mantel test was performed to determine the effects of geochemical factors on prokaryotic community composition (Smouse et al., 1986). The relationships between microbial diversity and environmental factors were evaluated using canonical correspondence analysis (CCA). Variation partitioning analysis (VPA) was employed to identify the relative importance of each environmental variable and their interactions in influencing the variation of prokaryotic communities (R Development Core Team, 2008). LEfSe (linear discriminant analysis coupled with effect size measurements) analysis was performed using the Kruskal–Wallis rank sum test to detect microbial taxa with significantly different abundances between the three sea areas, and LDA (linear discriminant analysis influence) was used to estimate the effect size of each taxon (Segata et al., 2011). To test the correlations between microbial community diversity and environmental factors, we adopted Spearman correlation analysis, which was performed with the R software (V 2.15.3).

### Statistical Analysis

Geochemical variables were analyzed using SPSS 19.0. The significant differences between selected geochemical variables and prokaryotic diversity were determined using one-way ANOVA (^∗^*P* < 0.05, ^∗∗^*P* < 0.01). The OTU significance by category command in QIIME was used to test for correlations between OTU abundance and geochemical factors using either the analysis of variance (ANOVA) test. *P-*values adjusted by Bonferroni’s correction were used to show statistical significance in OTU abundance correlation tests performed in QIIME.

## Results

### Environmental Characterization of Sediments

Generally, the contents of TC, TOC, and TN differed among the three regions, although these differences did not reach statistical significance. The TC contents of Prydz Bay were higher than those of the Antarctic Peninsula and Ross Sea samples, with average TC contents of 0.86 ± 0.5%, 0.63 ± 0.32% and 0.72 ± 0.31%, respectively. Similar, TN average content of the three region samples were 0.14 ± 0.05%, 0.1 ± 0.05%, and 0.12 ± 0.06%, respectively. However, TOC average content of Ross Sea samples was higher than those of the Prydz Bay and Antarctic Peninsula samples, with average contents of 0.54 ± 0.21%, 0.49 ± 0.27%, and 0.48 ± 0.21%, respectively (Table 2).

**TABLE 2:** Geochemical parameters of the 19 surface sediment samples from three regions of the Southern Ocean.

Sampling region	Sample name	Sample geochemical factors characteristic															
		TN (%)	TC (%)	TOC (%)	TFe_2_O_3_ (mg/kg)	P_2_O_5_ (mg/kg)	Al_2_O_3_ (mg/kg)	CaO (mg/kg)	MgO (mg/kg)	K_2_O (mg/kg)	Na_2_O (mg/kg)	MnO (mg/kg)	TiO_2_ (mg/kg)	Ba (μg/kg)	Sr (μg/kg)	V (μg/kg)	Zn (μg/kg)
Prydz Bay	P3.5	0.17	0.98	0.63	2.85	0.08	5.72	1.2	1.65	1.67	4.39	0.03	0.34	620	117	50.56	88.35
	P3.8	0.17	1.59	0.74	3.48	0.13	8.3	3.87	1.24	2.38	2.71	0.06	0.48	599	223	47.65	73.81
	P3.9	0.18	1.12	0.49	3.25	0.11	8.09	1.12	1.28	2.56	3.2	0.05	0.58	697	127	50.94	64.95
	P7.7	0.08	0.25	0.26	6.33	0.15	12.32	1.9	2.64	3.13	3.4	0.11	0.75	1787	190	87.06	142.23
	P7.9	0.05	0.27	0.21	7.17	0.14	13.05	1.78	2.73	3.13	3.15	0.07	0.77	889	181	99.18	115.05
	P7.16	0.18	0.95	0.65	3.37	0.12	7.15	1.59	1.82	1.76	4.76	0.04	0.44	631	140	54.1	81.63
Antarctic Peninsula	D2.6	0.04	0.28	0.19	7.16	0.17	13.64	5.51	3.38	1.46	3.41	0.1	0.79	325	332	176.12	87.63
	D1.7	0.14	0.86	0.65	7.31	0.23	13.37	3.42	3.49	1.64	4.81	0.11	0.81	378	238	156.61	108.92
	D5.4	0.07	0.54	0.41	3.58	0.15	10.06	1.67	1.55	1.74	3.14	0.05	0.56	393	158	76.87	64.32
	D5.5	0.18	1.03	0.86	2.37	0.11	6.13	1.18	1.39	1.08	3.98	0.04	0.35	303	107	51.37	58.72
	D5.6	0.07	0.42	0.29	1.68	0.07	5.47	1.06	0.88	1.35	2.85	0.02	0.35	336	116	38.36	35.99
Ross Sea	R01	0.05	0.35	0.26	3.44	0.17	12.45	2.61	1.47	2.94	3.41	0.07	0.56	703	256	48.1	59.76
	R02	0.09	0.55	0.46	7.16	0.33	12.45	3.11	1.8	2.96	4.56	0.09	1.07	456	280	71.34	129.59
	R04	0.15	0.83	0.63	4.29	0.21	9.62	2.38	2.03	2.2	4.83	0.07	0.75	628	235	69.08	97.68
	R05	0.18	0.97	0.74	3.51	0.14	7.25	1.51	1.67	1.74	4.16	0.05	0.47	594	146	54.21	97.59
	R08	0.02	0.19	0.19	3.68	0.11	11	2.49	1.58	2.67	2.88	0.06	0.48	531	216	56.51	50.64
	R10	0.14	0.73	0.6	3.72	0.14	8.42	2.03	1.87	1.91	3.87	0.05	0.57	588	178	62.93	93.43
	R11	0.17	0.97	0.62	3.31	0.13	8.2	1.75	1.7	1.88	3.93	0.06	0.5	613	156	59.48	91.37
	R17	0.19	1.13	0.81	2.86	0.12	6.63	1.34	1.66	1.64	4.66	0.03	0.43	556	130	54.82	94.63

Environmental factors such as Al, Fe, Mg, Ca, K, Na, Mn, Ti, V, Zn, and P contents were also compared with the different regions (Table 2), but their differences did not reach statistical significance. However, the Ba ion average content in the Antarctic Peninsula region samples (347.07 ± 37 μg/kg) was significantly lower than those in the Prydz Bay (870.48 ± 461 μg/kg, *P* = 0.005) and Ross Sea (583.62 ± 72 μg/kg, *P* = 0.001) samples. The K average content in the Antarctic Peninsula region samples (1.45 ± 0.25 mg/kg) was significantly lower than those in the Prydz Bay (2.43 ± 0.63 mg/kg, *P* = 0.007) and Ross Sea (2.24 ± 0.54 mg/kg, *P* = 0.017) samples.

### Prokaryotic Diversity Analysis

After the data were filtered, 1,079,709 clean tag sequences were obtained from the 19 sediment samples for further analysis, with an average of 56,826 ± 18,896 tag sequences per sample (Table 3). In total, 43,227 OTUs were obtained at a cutoff level of 97%, of which 41,671 (96.4%) could be classified, and the rarefaction curves of all stations reached asymptotes, which suggests satisfactory coverage in all samples (Supplementary Figure S1). In addition, the Good’s coverage values of all libraries were ≥96% (Table 3), indicating that the libraries could well reflect the bacterial communities of the samples.

**TABLE 3:** Biodiversity indices of the 19 surface sediment samples from three regions of the Southern Ocean.

Region	Sample ID	Raw tags	Tags (Nochime)	OTU	Observed species	Shannon	ACE	Coverage (%)
Prydz Bay	P3.5	87,664	85,434	5,767	999	6.07	2,389.86	0.97
	P3.8	57,547	55,610	4,549	980	6.67	1,803.82	0.98
	P3.9	57,845	56,299	3,732	1,059	6.89	2,479.36	0.97
	P7.7	84,374	81,012	7,057	1,293	7.53	3,130.64	0.97
	P7.9	88,703	86,419	6,311	1,162	7.74	2,279.60	0.97
	P7.16	76,795	74,618	5,473	1,044	6.44	2,710.03	0.97
Antarctic Peninsula	D2.6	32,042	27,609	972	894	6.14	1,766.49	0.98
	D1.7	32,806	27,019	628	512	4.09	2,276.90	0.98
	D5.4	38,106	33,031	1,010	1,111	6.04	3,100.31	0.96
	D5.5	40,881	34,915	826	651	4.50	1,447.76	0.98
	D5.6	41,712	37,646	1,123	1,061	7.01	2,201.80	0.98
Ross Sea	R01	67,321	62,952	536	257	2.74	734.71	0.99
	R02	58,958	53,383	856	601	5.70	1,368.86	0.98
	R04	73,857	67,632	826	474	4.49	1,384.98	0.99
	R05	44,484	40,623	619	414	4.54	1,179.43	0.99
	R08	67,977	60,363	541	287	3.54	727.37	0.99
	R10	70,513	64,415	727	415	4.19	1,220.23	0.99
	R11	69,309	61,563	838	513	4.99	1,435.09	0.98
	R17	75,109	69,166	836	453	4.35	1,240.33	0.99

The Shannon index was chosen to reflect the degree of bacterial community diversity; a greater Shannon index indicates higher diversity of the bacterial community (Liu et al., 2017). In our results, the Shannon index showed that the Prydz Bay sediments had the highest diversity, ranging from 6.07 to 7.74 (6.89 ± 0.64), followed by the Antarctic Peninsula region samples, ranging from 4.09 to 7.01 (5.56 ± 1.22, *P* = 0.029), whereas the lowest values of this index were found in the Ross Sea samples, ranging from 2.74 to 5.70 (4.37 ± 0.89, *P* = 0.000). The observed_species results showed the same trend, whereby the Prydz Bay sediments had the highest diversity, ranging from 980 to 1,293 (1089.5 ± 118), followed by the Antarctic Peninsula region sediments, ranging from 512 to 1,111 (845.8 ± 259, *P* = 0.026), and finally the Ross Sea sediments, ranging from 257 to 601 (426.75 ± 112, *P* = 0.000). The ACE indices showed the same trend, the Prydz Bay sediments was the highest, followed by Antarctic Peninsula region and Ross Sea (Table 3).

### Prokaryotic Relative Abundance and Community Composition Analysis

**FIGURE 2:**
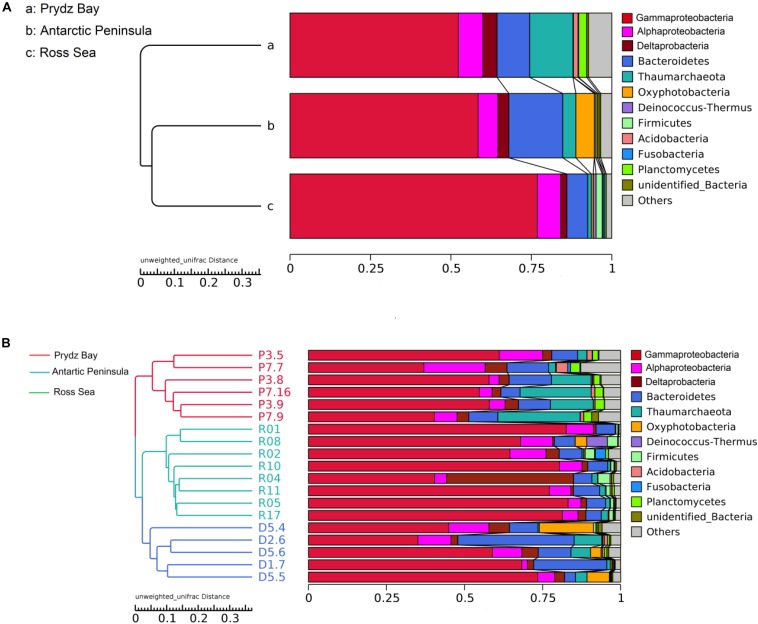
Clustering of surface sediment prokaryotic communities of three regions **(A)** and of each sample **(B)** by Unweighted Pair-group Method with Arithmetic Mean.

There were obvious differences in the archaeal relative abundance of these three distant regions. The highest archaeal relative abundance was found in the Prydz Bay sediments, where the average value was 0.14 (0.02–0.26), but the values were significantly lower in the Antarctic Peninsula region and Ross Sea sediments, at average 0.04 (0.004–0.09, *P* = 0.001) and 0.01 (0.0002–0.02, *P* = 0.000), respectively (Supplementary Figure S2). The prokaryotic community compositions were similar among the different regions, but the relative abundances were different; the dominant phylum was Proteobacteria, which comprised approximately 64% (51–78%) of total sequences in the Prydz Bay samples, 69% (48–82%) in the Antarctic Peninsula samples, and 86% (79–92%) in the Ross Sea samples, of which the most abundant was Gammaproteobacteria, followed by Alphaproteobacteria and Deltaproteobacteria at class level (Figure 2). The second most abundant phylum was Bacteroidetes, which accounted for approximately 10% (6–14%) of the Prydz Bay samples, 16% (3–37%) of the Antarctic Peninsula samples, and 6% (5–8%) of the Ross Sea samples. The third most abundant phylum was Thaumarchaeota, which comprised approximately 13% (3–26%) of the Prydz Bay samples, 3% (0.4–9%) of the Antarctic Peninsula samples, and 1% (0.03–2%) of the Ross Sea samples.

**FIGURE 3:**
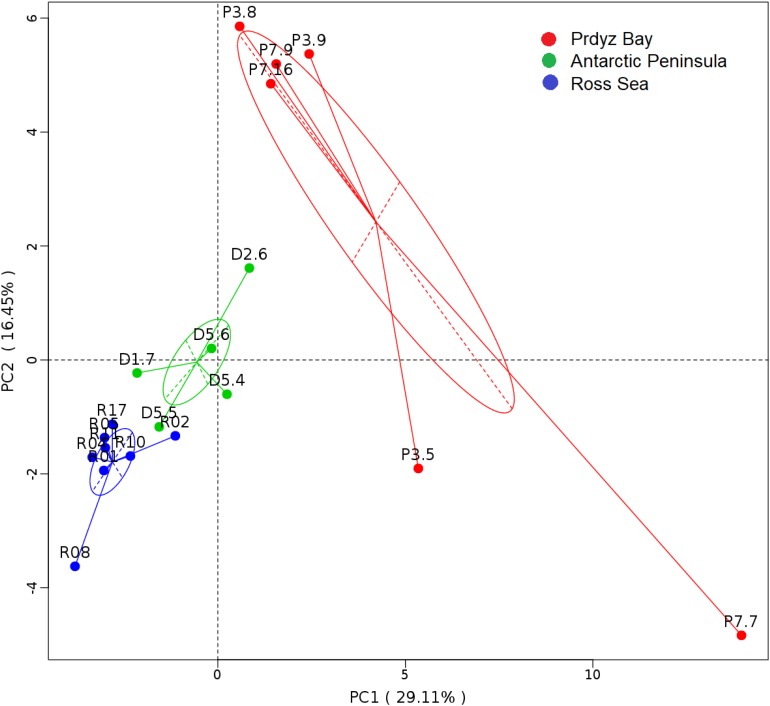
Principal coordinate analysis plots. The first principal coordinate (PC1) clearly separate samples by the location from which they were collected, as indicated by the ovals around pooled samples from the same collection site.

Cluster analysis showed that the 19 samples could be grouped into three categories according to their prokaryotic community compositions, which were in accordance with the sampling regions (Figure 2). Similar, the results of principal coordinate analysis support our results from UPGMA clustering when OTU are pooled by location (Figure 3). When sediment samples are pooled by location, the first principal coordinate (29.11%) separates samples based on the geographic locale from which they were sampled. The PCoA results also supported this cluster pattern (Supplementary Figure S3).

### Prokaryotic Community Differences Between the Three Sampling Regions

**FIGURE 4:**
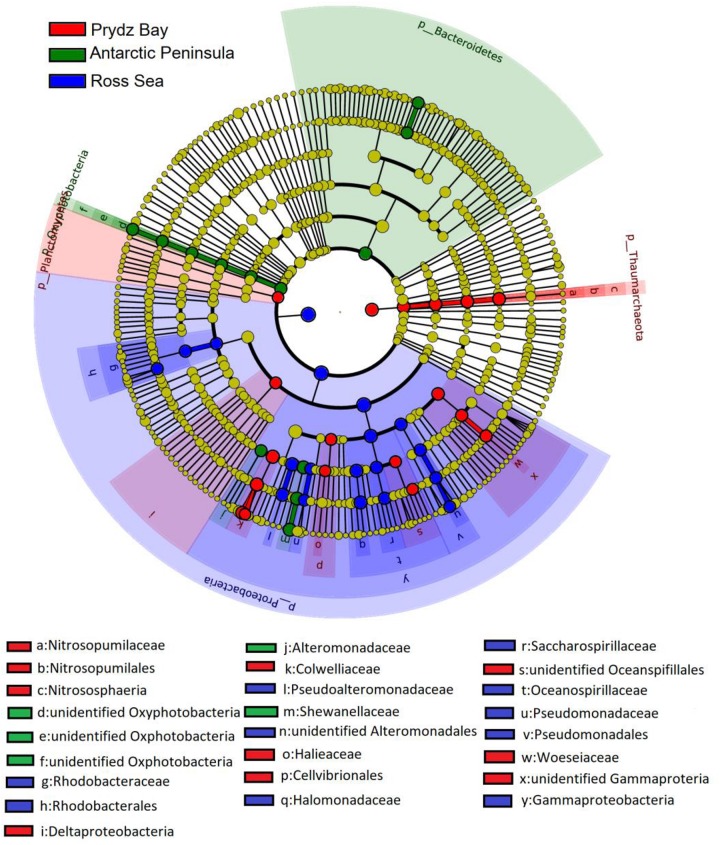
Identification of the distinct bacterial taxa from different sampling regions using LEfSe. Only bacterial taxa with LDA values greater than 4 are displayed in this cladogram. Differences in the most abundant taxa are represented by colors (red, green, and blue indicate Prydz Bay, the Antarctic Peninsula region, and the Ross Sea, respectively). The diameter of each circle is proportional to the corresponding taxon’s abundance. Circles represent phylogenetic levels from phylum to genus. Labels are shown from phylum to genus level.

In general, the prokaryotic community compositions were similar within the same sampling region but unique prokaryotic community was detected in each of the geographical regions. At the class level, Oxyphotobacteria had higher abundance in the Antarctic Peninsula samples, however, they were almost absent in the other two regions. However, the phylum Deinococcus–Thermus was observed only in the Ross Sea samples, and was absent in samples from the other two regions (Figure 4).

The LDA scores showed that bacterial community abundances also varied between the distant regions. Nineteen clades were found to have higher abundance in the bacterial community (LDA score > 4) of the Ross Sea: they mainly belonged to Proteobacteria, including Gammaproteobacteria, such as Pseudoalteromonadaceae, Alteromonadales, Oceanospirillales, and Rhodobacterales (Alphaproteobacteria). However, there were 13 clades with higher abundance in the bacterial community of the Antarctic Peninsula region, in where the high abundance of Bacteroidetes, such as *Flavobacterium*, *F. frigidarium* was found. In the Prydz Bay samples, there were 18 clades with higher abundance in the prokaryotic community, different with above, high abundance Archaea, such as Thaumarchaeota, Nitrosopumilaceae, Nitrosopumilales, Halieaceae, *Nitrososphaeria*, were found in this region (Supplementary Figure S4).

### Correlation With Environmental Factors

**FIGURE 5:**
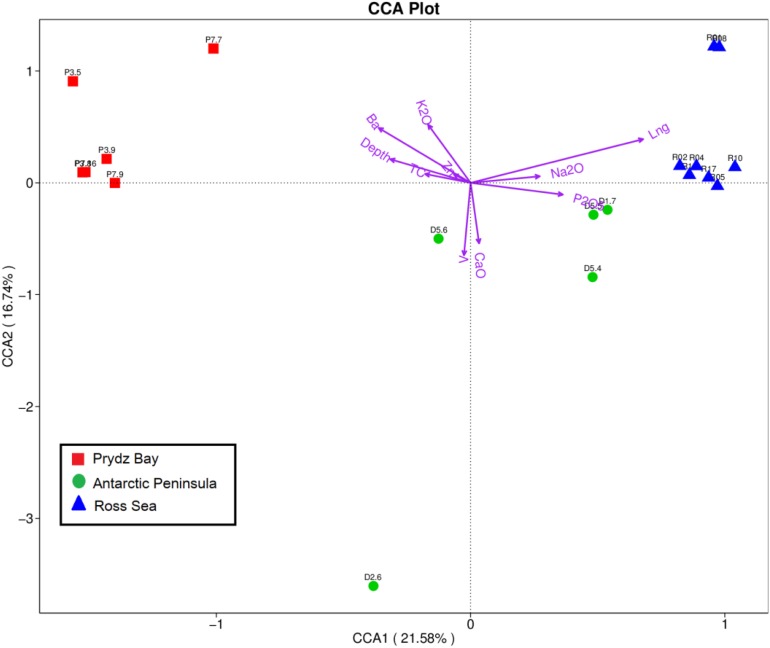
Canonical correspondence analysis diagram illustrating the relationships between the OTU-level community structures of different sampling sites. The angle between the arrow line and the sorting axis represents the correlation between a certain environmental factor and the sorting axis. The smaller angle represents the higher the correlation is, on the contrary, the lower the correlation is.

Geophysicochemical factors including geographical distance, water depth, and TC, Ca, P, Ba, Na, K, V, and Zn contents were correlated with prokaryotic community composition, screened based on the variance inflation factor (VIF). The Mantel test revealed that environmental factors (K, Na, Ba, V, and Zn contents, geographical distance, and water depth) were significantly correlated with prokaryotic community composition (*r* = 0.536, *p* = 0.001), with specific results as follows: V (*r* = 0.5073, *p* = 0.005), Ba (*r* = 0.4332, *p* = 0.007), geographical distance (*r* = 0.2787, *p* = 0.007), water depth (*r* = 0.2452, *p* = 0.064), Zn (*r* = 0.1028, *p* = 0.206), K (*r* = 0.0726, *p* = 0.214), and Na (*r* = 0.03315, *p* = 0.305). The nutrition factors (*r* = 0.179, *p* = 0.105), including TC (*r* = 0.1765, *p* = 0.063), Ca (*r* = 0.1658, *p* = 0.126), and *P* (*r* = 0.1271, *p* = 0.789), also showed different correlations with prokaryotic community composition. Subsequently, the relationships between the environmental factors and microbial diversity were analyzed by CCA. Environmental variables in the first two CCA dimensions explained 21.58% (CCA1) and 16.74% (CCA2) of the variance in the sediment microbial communities, respectively (Figure 5).

VPA was subsequently employed to calculate the relative importance of each factor in shaping variations in the bacterial communities. Nutrient factors (TC, P, and Ca) explained 13.4% of the observed variations, and other environmental factors explained 52.57%, leaving 26.72% of the variation unexplained. The environmental factors of geographical distance, Zn, K, water depth, Na, Ba, and V, respectively, explained 12.52, 11.73, 7.13, 5.83, 5.28, 2.53, and 2.52% of the variation, respectively, whereas the nutrient factors of P, Ca, and TC contents, respectively, explained 6.41, 5.96, and 3.62% of the observed variation. Additionally, an interaction was observed between nutrient factors and geophysicochemical factors that accounted for 7.31% of the variation. Therefore, geographical distance had the strongest effect among the evaluated factors on the spatial distribution of the prokaryotic communities.

### Effect of Environmental Factors on Prokaryotic Diversity and Community Composition

**FIGURE 6:**
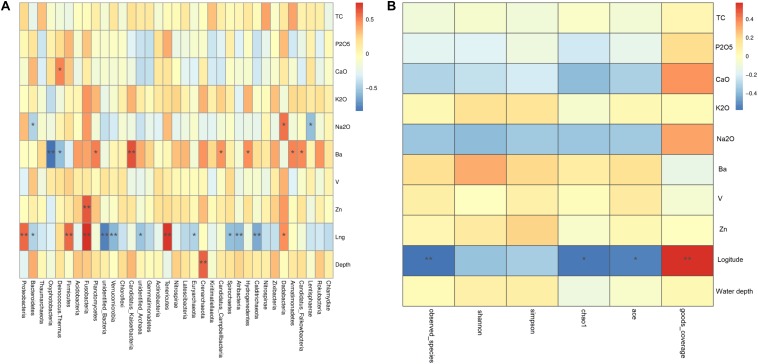
Spearman analysis of the effects of geochemical parameters on the phylogenetic structure of the prokaryotic communities. **(A)** Cluster analysis between main environmental factor and community abundance; **(B)** cluster analysis between main environmental factor and diversity indices. In Spearman correlation analysis the intermediate heat map corresponding value is the Spearman correlation coefficient r, r > 0 is the positive correlation, *r* < 0 is negative correlation, * indicates the significance test *P* < 0.05, ** indicates the significance test *P* < 0.01.

Similar with the above analysis, Spearman results showed that environmental factors have influence not only on prokaryotic diversity but also community composition, especially geographical distance and Ba content. First, geographical distance was found to be significantly positively related to the abundances of prokaryotic clades such as Dadabacteria, Tenericutes, Fusobacteria, Firmicutes, and Proteobacteria, but significantly negatively related to the abundances of prokaryotic clades such as Calditrichaeota, Atribacteria, Spirochaetes, Euryarchaeota, unidentified Archaea, Verrucomicrobia, unidentified Bacteria, and Bacteroidetes. And, Candidatus Falkowbacteria, Armatimonadetes, Hydrogenedentes, Candidatus Campbellbacteria, Candidatus Kaiserbacteria, and Planctomycetes were significantly positively related to Ba content, but Deinococcus–Thermus and Oxyphotobacteria were significantly negatively related to Ba content (Figure 6A). Figure 6B showed that geographical distance was found to be significantly positively related to goods_coverage indices, but significantly negatively related to ace indices, chaol1 indices and observed_species. Ba content was positively related to Shannon indices, simpson indices, ace indices, chaol1 indices, and observed_species, but these differences did not reach statistical significance. Therefore, geographical distance had significantly effect on the spatial distribution of the prokaryotic diversity and community composition.

## Discussion

HTS technology has been extensively used to elucidate the microbial diversity and community composition in various environments, such as soils, fresh water, and surface seawater. However, few studies using HTS technology have been reported on bacterial and archaeal diversity in deep-sea sediments in different region’s of the Southern Ocean (Bowman et al., 2000, 2003; Powell et al., 2003; Purdy et al., 2003).

The Southern Ocean is characterized by strong seasonality in environmental conditions, such as ice cover, mixed layer depths, light levels, and temperature, which have direct implications on microbial diversity and community structure (Smetacek and Nicol, 2005; Doney et al., 2012; Grzymski et al., 2012; Fuhrman et al., 2015; Bunse and Pinhassi, 2017). Previous studies have revealed that some microorganisms exhibit distinct biogeographical patterns (Horner-Devine et al., 2004; Green and Bohannan, 2006; Martiny et al., 2006), which appear to be controlled by differences in environmental variables in some cases (Horner-Devine et al., 2004), and by geographical distance in others (Cho et al., 2000; Whitaker et al., 2003). In general, our results showed that Prydz Bay had the greatest prokaryotic diversity, followed by the Antarctic Peninsula region, with the lowest prokaryotic diversity in the Ross Sea samples. Typically, the variability in diversity structures may be attributed to variable organic matter content (Takebayashi et al., 2007; Bell et al., 2013; Ji et al., 2015). Consistent with the results for diversity, the TOC content of the Prydz Bay samples (0.54 ± 0.21%) was higher than those of the Antarctic Peninsula and Ross Sea samples (0.49 ± 0.27% and 0.48 ± 0.21%), however, there were no obvious statistical differences in organic matter contents between the three regions. Aside from the organic matter content, other environmental factors shaped the relative abundance and biodiversity of microbial taxa. Our results showed similar bacterial communities, with Proteobacteria, Bacteroidetes, Thaumarchaeota, and Oxyphotobacteria observed in samples from all three regions, but with substantial variations in abundance between the different regions. Thaumarchaeota had the highest relative abundance (up to 13%) in the Prydz Bay samples, whereas Bacteroidetes and Oxyphotobacteria had the highest relative abundance in the Antarctic Peninsula samples, with up to 16 and 4.8% relative abundance, respectively. However, the highest relative abundance of Proteobacteria, up to 86%, was found in Prydz Bay. Recently, studies have increasingly demonstrated biogeographical distributions of marine microorganisms (Martiny et al., 2006; O’Malley, 2008; Galand et al., 2010; Schauer et al., 2010; Zinger et al., 2011). These results imply that spatial isolation affects community composition, presumably because of dispersal limitations (Xiong et al., 2014), especially between regions at great distances and with distinct hydrological surroundings (Jones et al., 1995; Jones, 2001; McLaughlin et al., 2004).

Microbial communities and their associated metabolic activities in marine sediments have important impacts on global biogeochemical cycles, but geophysicochemical factors have profound effects on microbial community composition and structure. In this study, we show significant statistical relationships between variations in geophysicochemical factors and prokaryotic community structure in sediments between three regions. Geographical distance and Ba content were found to statistically significantly affect prokaryotic community variation at the phylum level. The higher abundance of Proteobacteria and lower relative abundance of Bacteroidetes were found in the Ross Sea samples, in contrast, a higher relative abundance of Thaumarchaeota was found in the Prydz Bay samples than in the Antarctic Peninsula and Ross Sea samples, probably related with the longitude variation of sample collected location (geographical distance). These results are in agreement with previous studies, which indicated that geographical distance is a potential factor impacting bacterial community composition and distribution (Martiny et al., 2006; O’Malley, 2008). Besides, the higher relative abundance Archaea was found in Prydz Bay samples probably due to the deeper water depth of sampling site, especially P3.5 (2064 m) and P3.8 (1487 m) (Supplementary Figure S2). Similar, the relative abundance of Crenarchaeota was significantly positive related to water depth (Figure 6A), previous studies have found that heterotrophic bacterial abundance declined significantly with water depth (Arístegui et al., 2009).

Second, Ba content showed statistically significant correlation with prokayotic community variations. A previous study showed that the total Ba ion concentration of Prydz Bay sediment was 513–874 μg/kg, with an average value of 654 μg/kg (Tan et al., 2014). Our results showed Ba ion concentrations of 599–1,787 μg/kg, with an average value of 870.48 ± 461 μg/kg, higher than the previous report possibly because the sampling sites differed. In the previous study, it was speculated that the Ba ion content was positively related to high primary productivity in the ocean. Planctomycetes were more abundant in Prydz Bay than in the other two regions, which is in accordance with the higher Ba ion content in the Prydz Bay samples. However, Oxyphotobacteria were more abundant in the Antarctic Peninsula region than in the other two regions (Figure 6A). This result implies that Ba ion content may have an important effect on microbial community structure, such as, higher relative abundance Archaea, especially Thaumarchaeota, were found in Prydz Bay, but more evidence is still needed to support this hypothesis. Previous studies have shown that the iron and manganese contents in sediments are strongly related to microbial community structure (Biddle et al., 2006; Orcutt et al., 2011), but a relationship between Ba ion contents and microbial community structure has not previously been reported. However, V ion content was not significantly correlated with bacterial community distribution at the phylum level in this region, although it showed a significant effect on bacterial diversity in the VIF analysis. Similar to previously reported results (Biddle et al., 2006; Orcutt et al., 2011), our results suggest that mineralogy is strongly related to microbial community structure.

Microbial organisms are ubiquitously distributed in marine environments and play pivotal roles in maintaining ecosystem functions. In addition, these microbial organisms have distinct geographical distributions, which contribute significantly to biomass and primary production in their ecosystems. In this study, distinct prokaryotic community compositions were observed between the three sampling sites of the Southern Ocean. *Nitrososphaeria*, Nitrosopumilaceae, Nitrosopumilates, Thaumarchaeota, Deltaproteobacteria, Colwelliaceae, Halieaceae, and Planctomycetes are taxa distinct to Prydz Bay. Similarly, Wilkins et al. (2012) detected abundant OTUs of *Nitrosomonas europaea*, *Nitrosomonas eutropha*, and *Nitrosospira multiformis* strains in Southern Ocean surface waters by metagenomic analysis. Such bacteria of the Nitrospirae may also be nitrifiers, important in the processes of nitrite oxidation and carbon fixation in deep-sea environments (Ehrich et al., 1995). In addition, the ammonia-oxidizing Archaean of the Thaumarchaeota aerobically oxidize NH^4+^ to NO^2–^, and Nitrosospira completely oxidize NH^4+^ to NO^3–^ to conserve energy (Daims et al., 2015), which may play important roles in nutrient capture and the biogeochemical cycles of phosphorus and nitrogen. Studies have increasingly reported that members of the Thaumarchaeota are among the most important ammonia-oxidizing organisms and play important roles in the nitrogen cycles of marine environments (Francis et al., 2007; Mincer et al., 2007). Planctomycetes are characterized by a wide distribution in most marine environments (Fuerst, 1995; Polymenakou et al., 2009) and catalyze important transformations in the global carbon and nitrogen cycles (Glöckner et al., 1999). Planctomycetes have been proposed to exhibit unique biogeochemical properties such as anaerobic ammonium oxidation (Jasmin et al., 2017), methane oxidation (Bhattacharyya et al., 2017), and participation in carbon recycling (Woebken et al., 2007). Deltaproteobacteria have rarely been detected in abundance in surface waters (Venter et al., 2004), but were frequently encountered in sediments of Prydz Bay, in our result, Deltaproteobacteria was detected in the sediment of all three regions. The Deltaproteobacteria participate in carbon fixation via the Calvin cycle and in sulfur oxidation, as well as in oxidation of methylated compounds (Swan et al., 2011); therefore, members of this class may be significant contributors to chemoautotrophy in the dark ocean (Swan et al., 2011). In our results, the TN and TC contents of Prydz Bay were higher than those of the Antarctic Peninsula and Ross Sea, although the differences did not reach statistical significance. Thus, the high-abundance members of the prokaryotic community that participate closely in the carbon and nitrite recycling processes may be correlated with the higher contents of TN and TC in the Prydz Bay samples.

Oxyphotobacteria, Bacteroidetes, *Flavobacterium*, Alteromonadaceae, and Shewanellaceae were the main distinct taxa in the Antarctic Peninsula region. Oxyphotobacteria (also known as blue-green bacteria) are important primary producers, with some taxa capable of fixing both atmospheric carbon and nitrogen (Hartmann et al., 2014; Karlson et al., 2015). *Flavobacterium* is widespread in distribution and has been isolated from many habitats, including in Antarctica. This genus appears primarily to play a role in remineralization processes and exhibits strong macromolecular hydrolytic capabilities (McCammon and Bowman, 2000). These bacteria attach to phytoplankton aggregates and efficiently degrade and preferentially consume high-molecular-mass organic matter, rather than monomeric organic compounds, as primary carbon and energy sources (Ward, 1996). In our study, high abundances of Oxyphotobacteria and *Flavobacterium* were detected, which may be correlated with high abundance of phytoplankton in the Antarctic Peninsula region (Moor et al., 2002); as important primary producers, they contribute significantly to primary production in the Antarctic Peninsula region.

Rhodobacteraceae, Rhodobacterales, *Marinobacter*, *Sulfitobacter*, Pseudoalteromonadaceae, Pseudoalteromonadales, Oceanospirillales, *Halomonas*, and Gammaproteobacteria were the main distinct taxa in the Ross Sea samples. Many species of the Rhodobacteraceae are known for their close associations with algal blooms, as well as with organic particles (Cottrell and Kirchman, 2000; Pinhassi et al., 2004; Wagner-Döbler and Biebl, 2006). Indeed, many members of the Rhodobacteraceae are aerobic heterotrophs that preferentially use labile organic substrates (Cottrell and Kirchman, 2000), but may also perform photosynthesis in the presence of O_2_ or under anaerobic conditions (Brinkhoff et al., 2008; Elifantz et al., 2013; Koblížek et al., 2015). *Marinobacter* was previously isolated from marine algae (*Ulva fenestrata*) and has the ability to degrade proteins and complex polysaccharides (Nedashkovskaya et al., 2004). In our study, the TOC content of Ross Sea sediments was higher than those of the other two regions, although there were no obvious statistical differences between the three regions. The TOC contents of Ross Sea was 0.54 ± 0.21%, this results are similar to those of a previous study, which showed that TOC contents in Ross Sea sediment were 0.25–1.42%, with an average value of 0.38% (Chen et al., 2012). In addition, past studies showed that the TOC content of Ross Sea sediment mainly originated from marine algae, based on the TOC/TN ratio and δ^13^ C value of the sediment (Emerson and Hedges, 1988; Stein, 1991). High abundance of Rhodobacteraceae species and *Marinobacter* may be correlated with sufficient nutrients and phytoplankton community abundance in the Ross Sea (Chen et al., 2012). In addition, *Sulfitobacter* are autotrophic nitrite-oxidizing and heterotrophic sulfite-reducing bacteria. Gammaproteobacteria are also putative sulfur-oxidizers with the potential for autotrophic denitrification coupled with sulfur oxidation (Goffredi et al., 2004). Sulfur oxidation had been suggested to be the primary process of energy metabolism driving deep-sea vent ecosystems (Nakagawa et al., 2005).

## Conclusion

In conclusion, our study provides the first profiles of prokaryotic diversity and community composition in sediment samples from 19 sites in Prydz Bay, the Antarctic Peninsula region, and the Ross Sea based on HTS. Prydz Bay sediments showed the greatest prokaryotic diversity, followed by the Antarctic Peninsula sediments, with the lowest prokaryotic diversity found in the Ross Sea sediments. Proteobacteria, followed by Bacteroidetes, Thaumarchaeota, Oxyphotobacteria, and Deinococcus–Thermus, dominated the sediment prokaryotic communities in these regions of the Southern Ocean. However, distinct differentiation of prokaryotic communities was observed between the three sampling regions. Geophysicochemical factors, especially geographical distance and Ba ion content, significantly contributed to the prokaryotic diversity and community distribution of the Southern Ocean sediments.

## Data Availability Statement

The original contributions presented in the study are publicly available. This data can be found here: https://www.ncbi.nlm.nih.gov/sra, BioProject ID: PRJNA615046.

## Author Contributions

JL, XG, and YG collected samples, designed research, supervised the project, analyzed the data, and wrote the manuscript. XG performed the experiments, analyzed the data, and prepared the figures. YG performed clustering and PCA analysis, analyzed the data, and prepared figures.

## Conflict of Interest

The authors declare that the research was conducted in the absence of any commercial or financial relationships that could be construed as a potential conflict of interest.
