# Supplementary material for: Prokaryotic Diversity and Composition of Sediments From Prydz Bay, the Antarctic Peninsula Region, and the Ross Sea, Southern Ocean
Source: Front Microbiol. 2020 Apr 28;11:783. doi: 10.3389/fmicb.2020.00783 (PMC7198716; doi:10.3389/fmicb.2020.00783)
Supplement: Supplementary file 1 [file Data_Sheet_1.PDF]

Supplementary Figures

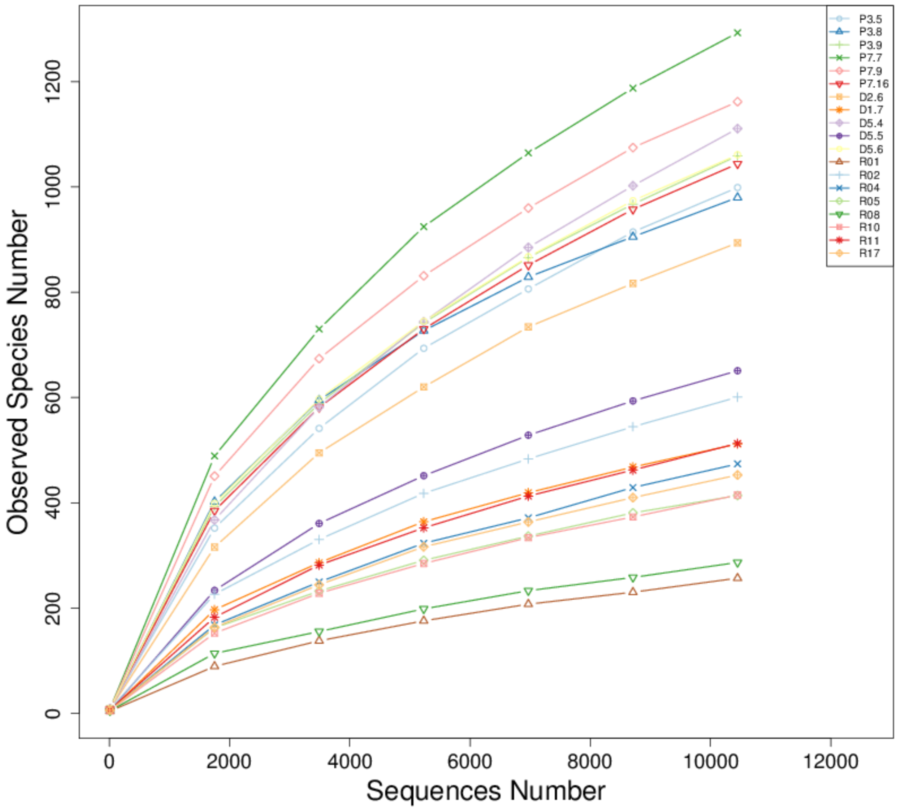

Supplementary Figure 1. Rarefaction curves of nineteen samples from the Southern Ocean.

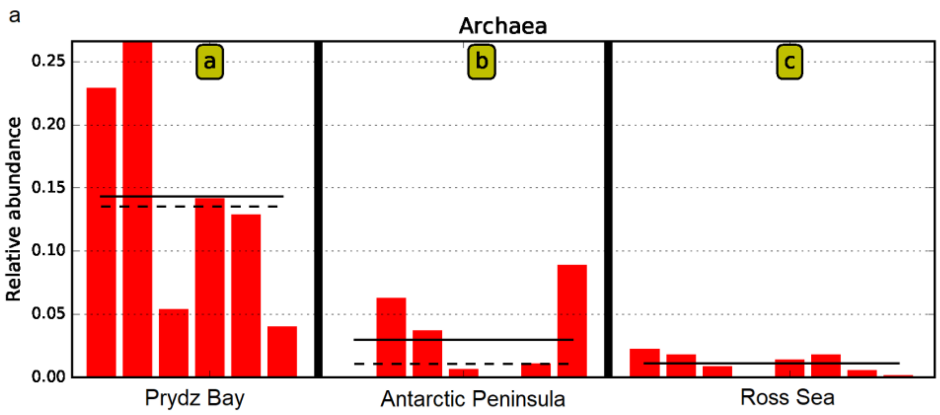

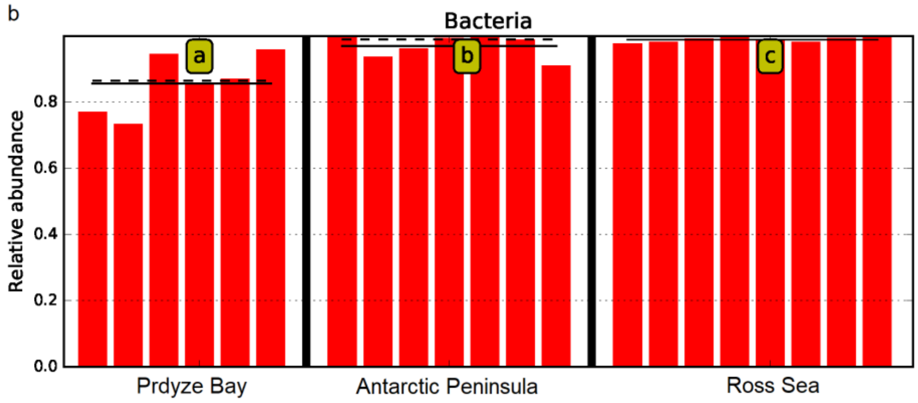

Supplementary Figure 2. Abundance of Archaea (a) and Bacteria (b) in 19 samples from the Southern Ocean.

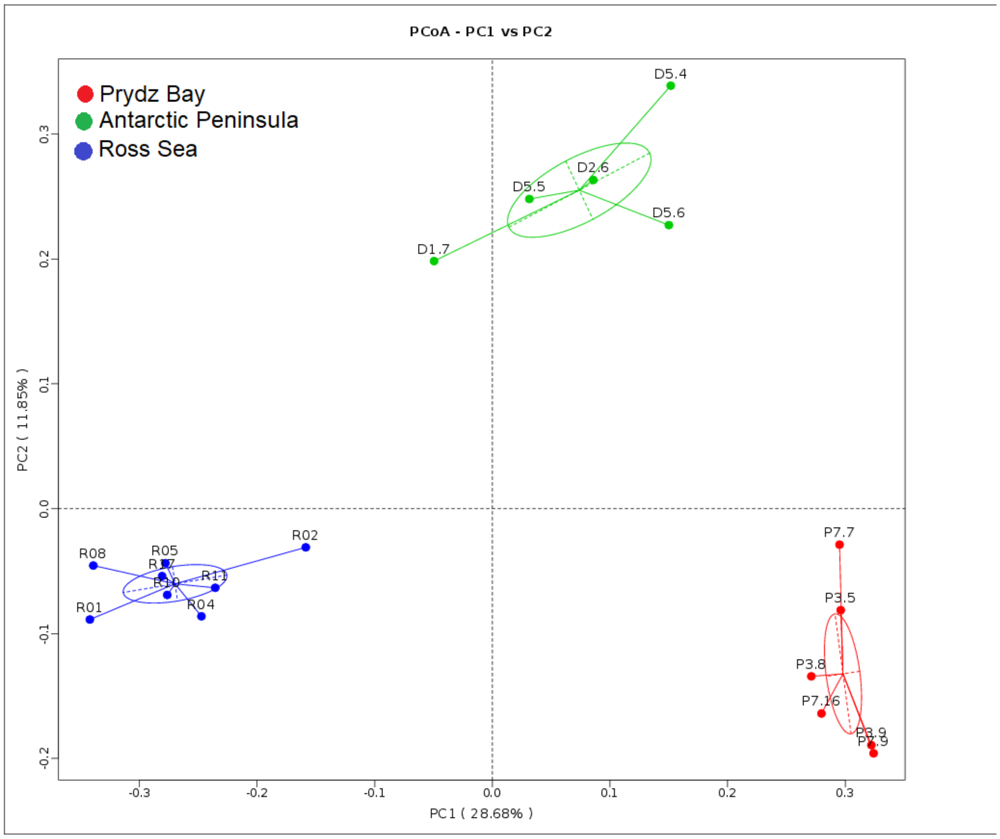

Supplementary Figure 3. PCoA of bacterial community compositions based on unweighted UniFrac distance.

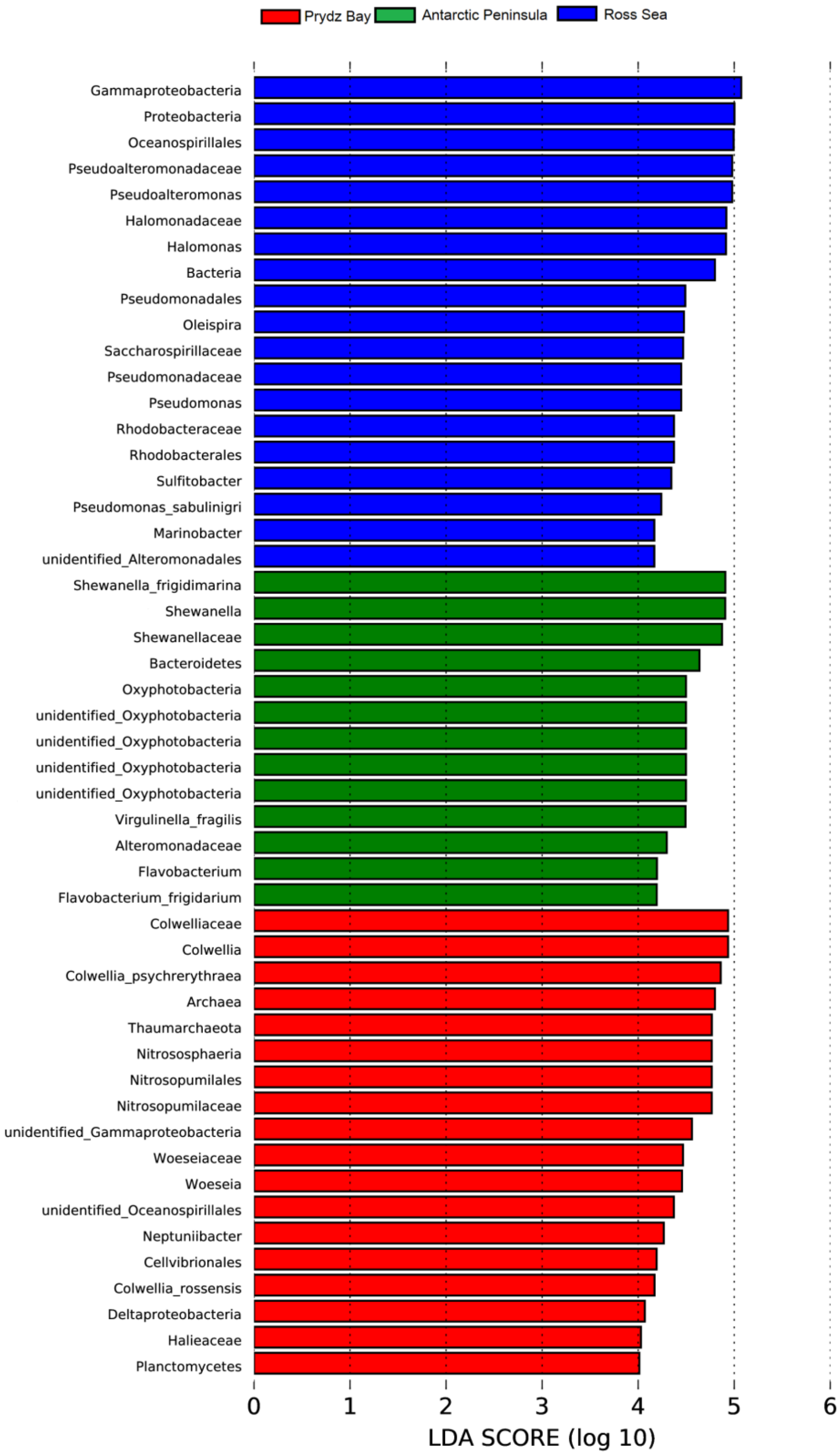

Supplementary Figure 4. LDA value distribution histogram showing bacterial communities with LDA scores greater than 4.
